# Redox Signaling and Regional Heterogeneity of Endothelial Dysfunction in *db/db* Mice

**DOI:** 10.3390/ijms21176147

**Published:** 2020-08-26

**Authors:** Nada A. Sallam, Ismail Laher

**Affiliations:** 1 Department of Pharmacology and Toxicology, Faculty of Pharmacy, Cairo University, Kasr Al-Aini Street, Cairo 11562, Egypt; nada.sallam@pharma.cu.edu.eg; 2 Department of Anesthesiology, Faculty of Medicine, Pharmacology and Therapeutics, The University of British Columbia, 2176 Health Sciences Mall, Vancouver, BC V6T 1Z3, Canada

**Keywords:** vascular heterogeneity, diabetes, redox signaling, oxidative stress, endothelial dysfunction, superoxide dismutase, *db/db* mice

## Abstract

The variable nature of vascular dysfunction in diabetes is not well understood. We explored the functional adaptation of different arteries in *db/db* mice in relation to increased severity and duration of diabetes. We compared endothelium-dependent and -independent vasodilation in the aortae, as well as the carotid and femoral arteries, of *db/db* mice at three ages in parallel with increased body weight, oxidative stress, and deterioration of glycemic control. Vascular responses to in vitro generation of reactive oxygen species (ROS) and expression of superoxide dismutase (SOD) isoforms were assessed. There was a progressive impairment of endothelium-dependent and -independent vasorelaxation in the aortae of *db/db* mice. The carotid artery was resistant to the effects of *in vivo* and in vitro induced oxidative stress, and it maintained unaltered vasodilatory responses, likely because the carotid artery relaxed in response to ROS. The femoral artery was more reliant on dilation mediated by endothelium-dependent hyperpolarizing factor(s), which was reduced in *db/db* mice at the earliest age examined and did not deteriorate with age. Substantial heterogeneity exists between the three arteries in signaling pathways and protein expression of SODs under physiological and diabetic conditions. A better understanding of vascular heterogeneity will help develop novel therapeutic approaches for targeted vascular treatments, including blood vessel replacement.

## 1. Introduction

The high morbidity and mortality rates in diabetic patients are largely due to cardiovascular complications [1]. Endothelial dysfunction is a key factor in diabetic vascular complications [2,3], as the endothelium regulates most aspects of vascular homeostasis including blood flow, blood clotting, angiogenesis, and inflammatory response [4]. Endothelium-dependent vasodilatation is an independent predictor of cardiovascular events and death in diabetic patients [5,6,7].

Although the majority of studies in diabetic subjects or animal models reported reduced endothelial-dependent vasodilatation [2,8,9], some showed unaltered responses [10,11,12] while others reported enhanced vasodilatation [13,14,15]. These discrepancies can be attributed to differences in species, diabetes severity, the signaling pathway examined, and/or different experimental methods. Even when using the same species and strain such as *db/db* mice (a commonly used model of type 2 diabetes), endothelium-dependent, acetylcholine (ACh)-induced vasodilatation was reduced in the aorta [16], mesenteric arteries [17], coronary artery [18], and coronary arterioles [19], but not in gracilis muscle arterioles [20]. It is plausible that different vascular beds serving specialized functions via specific signaling pathways function differently under physiological conditions, and they likely respond differently to pathophysiological stimuli. Diabetes and atherosclerosis target specific regions in the vasculature [21], even though all vascular beds are exposed to the same systemic abnormalities of hyperglycemia, oxidative stress, and hyperlipidemia associated with these diseases. 

There are several mechanisms that link hyperglycemia to vascular injury, including increased oxidative stress [22]. Elevated levels of biomarkers of oxidative stress were reported in diabetic patients [1,23] and animal models of diabetes [24,25,26]. There are several sources of reactive oxygen species (ROS) in the vasculature, including nicotinamide adenine dinucleotide phosphate oxidase (NADPH oxidase), cyclooxygenase (COX), uncoupled endothelial nitric oxide synthase (eNOS), and mitochondrial respiratory chain enzymes; ROS are generated by endothelial cells, vascular smooth muscle cells (VSMCs), and infiltrating neutrophils [27,28,29]. The presence of multiple sources of ROS suggests that ROS are essential cellular components that regulate vital functions via their interactions with potassium channels, calcium signaling, and various kinases [30,31,32,33,34]. 

This study compared endothelium-dependent and -independent vasodilatation of three conduit arteries: the aorta, as well as the carotid and femoral arteries, as related to deterioration in glycemic control and increases in oxidative burden in *db/db* mice at three ages. Importantly, these arteries were isolated from the same animal and examined under identical experimental conditions. Understanding these regional differences will improve our understanding of vascular homeostasis and guide the development of novel therapeutic approaches based on vascular heterogeneity.

## 2. Results

### 2.1. Age-Related Changes in Body Weight, Glycemic Control, and Systemic Oxidative Stress Biomarkers in db/db Mice

**Figure 1:**
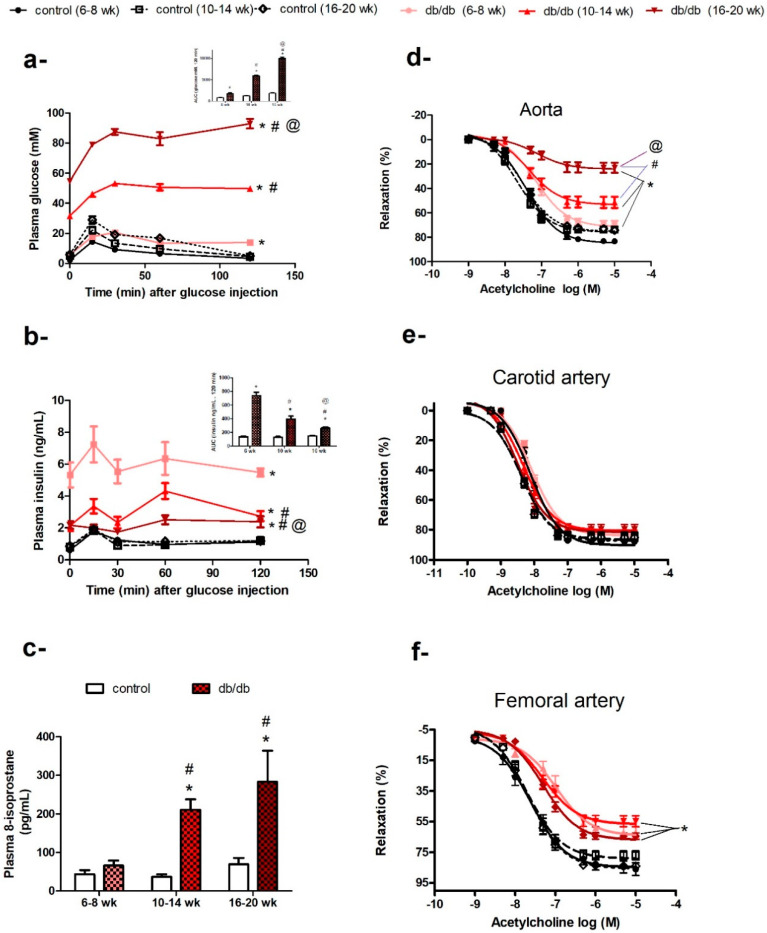
Age-related changes in diabetic status and endothelial dysfunction in *db/db* mice. Plasma glucose (**a**) and insulin (**b**) levels following an intraperitoneal injection of glucose in *db/db* and control mice at six, 10, or 16 weeks of age (*n* = 6–10; areas under the curve (AUCs) were compared by two-way ANOVA followed by Bonferroni post-test). (**c**) Histogram of plasma 8-isoprostane levels in *db/db* and control mice at six, 10, or 16 weeks of age (*n* = 5–10; two-way ANOVA followed by Bonferroni post-test). Cumulative concentration–response curves of acetylcholine in the aortae (**d**), carotid arteries (**e**), and femoral arteries (**f**) of *db/db* and control mice at 6–8 weeks, 10–14 weeks, and 16–20 weeks of age (*n* = 6–10 mice; two-way repeated-measures ANOVA followed by Bonferroni post-test). Results are expressed as mean ± standard error (SE). * denotes *p* < 0.05 vs. age-matched control, # denotes *p* < 0.05 vs. *db/db* (6–8 weeks), @ denotes *p* < 0.05 vs. *db/db* (10–14 weeks).

At the age of 6–8 weeks old, *db/db* mice weighed more than control mice (30.74 ± 0.37 g vs. 20.67 ± 0.34 g, *p* < 0.05). Although their fasting plasma glucose levels were not different from control mice (5.68 ± 0.31 mM vs. 4.36 ± 0.15 mM, *p* > 0.05), they exhibited impaired glucose tolerance (Figure 1a) and hyperinsulinemia (Figure 1b) compared to control mice. However, their plasma levels of 8-isoprostane were not significantly different from control mice (Figure 1c). 

As *db/db* mice got older (10–14 weeks old) and more obese (45.87 ± 0.65 g vs. 27.96 ± 0.29 g), they developed hyperglycemia (31.54 ± 1.33 mM vs. 5.03 ± 0.23 mM, *p* < 0.05), with a further deterioration in glucose tolerance (Figure 1a) and an elevation in plasma 8-isoprostane levels (a biomarker of systemic oxidative stress) (Figure 1c). When *db/db* mice were 16–20 weeks old, they had increased plasma 8-isoprostane levels, indicating higher levels of systemic oxidative stress (Figure 1c), and an age-related deterioration of glycemic control; the area under the curve for glucose (AUC_glucose_) for *db/db* mice increased from 477% to 546% (*p* < 0.05) compared to their age-matched controls (Figure 1a). Although, *db/db* mice remained hyperinsulinemic at all the ages studied, as indicated by their fasting insulin levels and AUC_insulin_, their ability to secrete insulin decreased from 540% (six weeks old) to 305% (10 weeks old) then 178.1% (16 weeks old), compared to age-matched control mice (Figure 1b). In contrast, control mice showed an age-dependent increase in body weight, but otherwise maintained steady levels for all other measured metabolic parameters. 

### 2.2. Age-Related Changes in Endothelium-Dependent Vasodilatation in Aortae but not in Carotid or Femoral Arteries of db/db Mice

As described above, *db/db* mice showed age-related increases in body weight, fasting plasma glucose, and plasma 8-isoprostane, with deterioration in glycemic control and insulin secretion reflecting the progression of metabolic dysfunction associated with diabetes. In parallel with the progression of diabetic status, the aortae of *db/db* mice (6–8 weeks old) exhibited a small but significant impairment in vasodilatory response to ACh, which deteriorated further with age in terms of maximum response (Emax: *db/db*_(6–8 weeks)_ = 71.59% ± 1.51%, *db/db*_(10–14 weeks)_ = 53.4% ± 2.06%, and *db/db*_(16–20 weeks)_ = 24.34% ± 1.84%, *p* < 0.05; Figure 1d), but not sensitivity (−log half maximal effective concentration (EC_50_): *db/db*_(6–8 weeks)_ = 7.1 ± 0.05 M, *db/db*_(10–14 weeks)_ = 7.3 ± 0.1 M, and *db/db*_(16–20 weeks)_ = 7.1 ± 0.2 M, *p >* 0.05; Figure 1d). However, the vasodilatory response to ACh was preserved in carotid arteries of *db/db* mice in the three age groups examined (Figure 1e). ACh-induced vasodilatation was impaired in the femoral arteries of *db/db* mice (6–8 weeks old); Emax: *db/db*_(6–8 weeks)_ = 64.18% ± 2.34% vs. control _(6–8 weeks)_ = 84.79% ± 2.24%, *p* < 0.05), but did not deteriorate further with age (Emax: *db/db*_(6–8 weeks)_ = 64.18% ± 2.34%, *db/db*_(10–14 weeks)_ = 56.75% ± 1.77%, *db/db*_(16–20 weeks)_ = 66.96 ± 1.2%, *p* > 0.05; Figure 1f). 

### 2.3. Preserved Endothelium-Dependent Vasodilatation in Carotid Arteries of db/db Mice in the Presence of Increased Oxidative Burden

**Figure 2:**
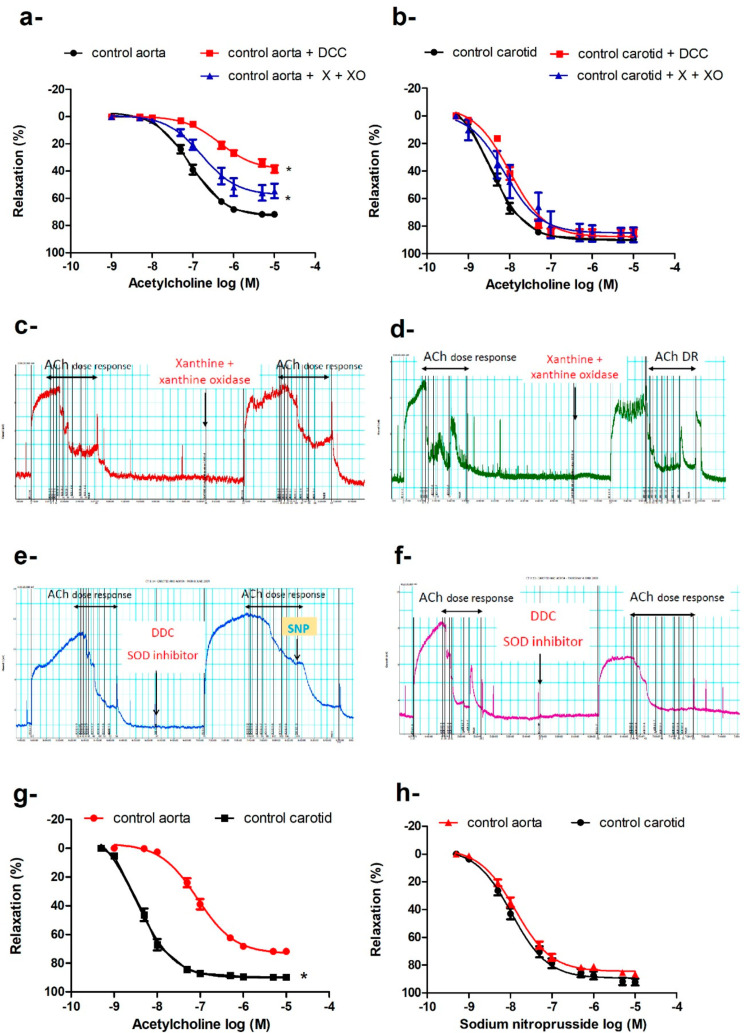
Differential response of the aorta and carotid artery to increased oxidative burden. Cumulative concentration–response curves of acetylcholine in aortae (**a**) and carotid arteries (**b**) in the presence or absence of xanthine and xanthine oxidase or diethyldithiocarbamate of 16–20-week-old control mice (*n* = 5–7 mice). Traces showing acetylcholine-induced vasodilatation in absence and presence of xanthine and xanthine oxidase in aorta (**c**) and carotid artery (**d**), and in absence and presence of DCC in aorta (**e**) and carotid artery (**f**) (for traces in panels c to f: x-axis is time (minutes) and y-axis is force (mN)). Cumulative concentration–response curves of acetylcholine (**g**) and sodium nitroprusside (**h**) in the carotid arteries and aortae of 16–20-week-old mice (*n* = 6–10 mice). Results are displayed as mean ± SE. Dose–response curves were compared by two-way repeated-measures ANOVA followed by Bonferroni post-test. * denotes *p* < 0.05 vs. control aorta. DCC = diethyldithiocarbamate, X = xanthine, XO = xanthine oxidase.

Despite increases in body weight, hyperglycemia, and oxidative stress in *db/db* mice, the vasodilatory responses to ACh were preserved in the carotid arteries of *db/db* mice in the three age groups (Figure 1e), in contrast to the progressive impairment of ACh-induced vasodilatation in the aortae (Figure 1d). We explored several possibilities that could underlie this differential adaptation. When carotid arteries and aortae of control mice were challenged with xanthine and xanthine oxidase as an exogenous source of ROS, the vasodilatation to ACh was impaired in the aortae (Emax: 57.75% ± 2.85% vs. 73.45% ± 1.27%, *p* < 0.05; −log EC_50_: 7.06 ± 0.04 M vs. 6.78 ± 0.12 M, *p* <0.05; Figure 2a,c), while the maximum vasodilator response in the carotid arteries remained unaffected (Figure 2b,d); there were minor reductions in sensitivity (−log EC_50_: 8.48 ± 0.04 M vs. 8.12 ± 0.15 M), indicating altered receptor-activated pathways. We also preincubated the aortic and carotid rings with diethyldithiocarbamate (DCC), an inhibitor of superoxide dismutase (SOD), to reduce the effects of the main endogenous antioxidant defense mechanism. Again, ACh-induced maximum vasodilatation was impaired in the aortae (Figure 2a,e) but not carotid arteries (Figure 2b,f). DCC did not impair sodium nitroprusside (SNP)-induced vasodilation in the aortae (Figure 2e), ruling out non-specific action of DCC on vasodilatory responses. 

Preincubation of the aortic or carotid rings with L-NAME (N omega-Nitro-L-arginine methyl ester) (10^−4^ M) abolished ACh-induced vasodilatation in *db/db* and control mice at all ages studied. The aorta and carotid artery show different sensitivity and efficacy for ACh, but not for SNP (Figure 2g,h). The carotid artery is more sensitive to ACh (−log EC_50_: carotid = 8.49 ± 0.05 M vs. aorta = 7.54 ± 0.06 M, *p* < 0.05) and relaxed to a greater extent (Emax: carotid = 90.25% ± 1.04% vs. aorta = 75.52% ± 1.4%, *p* < 0.05). There were no differences in sensitivity or efficacy for SNP between the carotid artery and the aorta. 

### 2.4. Signaling Pathway of Endothelium-Dependent Vasodilatation in the Carotid Artery

#### 2.4.1. Effects of Tempol, Catalase, or Ebselen on ACh-Induced Vasodilatation

**Figure 3:**
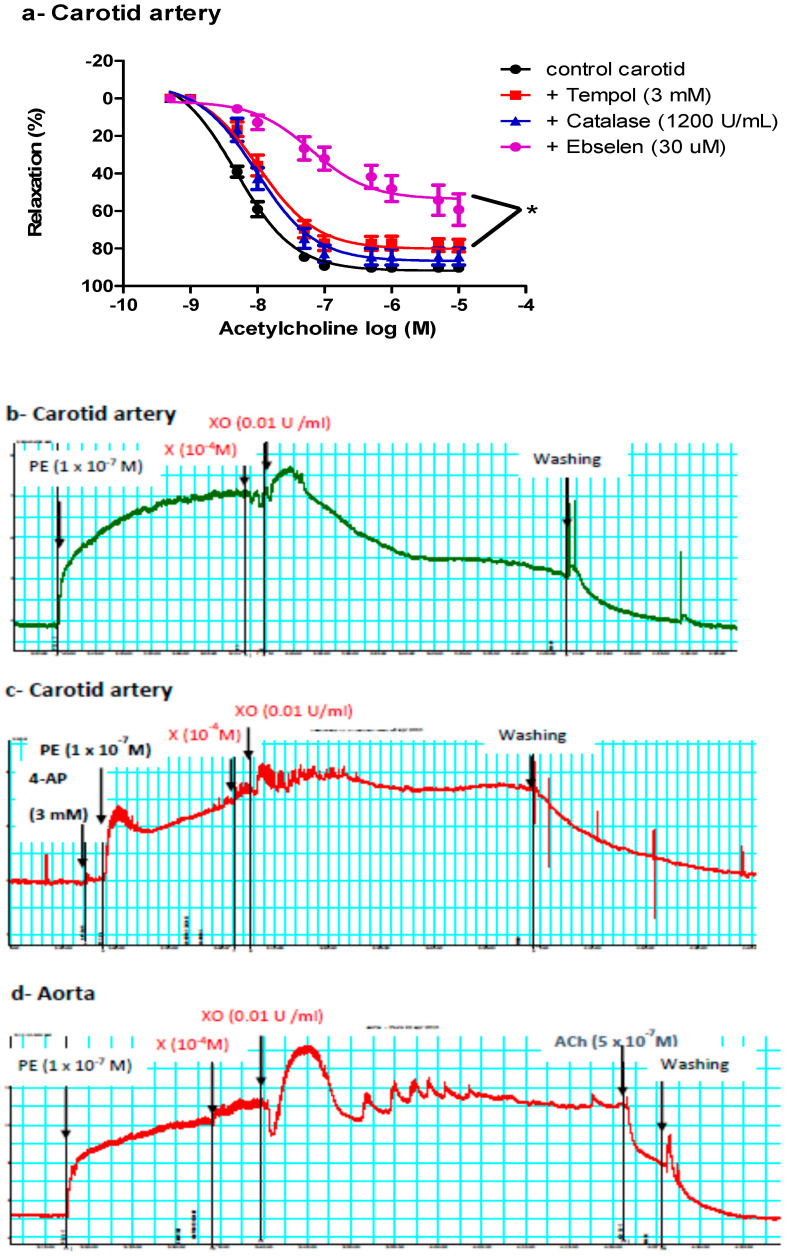
Role of reactive oxygen species (ROS) in mediating vasodilatation in the carotid artery. (**a**) Cumulative concentration–response curves of acetylcholine in the carotid arteries of control and *db/db* mice (14–20 weeks old) in the presence or absence of tempol, catalase, or ebselen (*n* = 5–10 mice). (**b**) A representative trace showing vasodilatation after adding xanthine and xanthine oxidase to the carotid artery (*n* = 16 rings from six mice). (**c**) A representative trace showing inhibition of the vasorelaxant effect of xanthine and xanthine oxidase in the carotid artery in the presence of 4-aminopyridine (*n* = 4 from four mice). (**d**) A representative trace showing no vasodilatation after adding xanthine and xanthine oxidase to the aorta (*n* = 9 rings from five mice). For traces in panels b, c and d: x-axis is time (minutes) and y-axis is force (mN). 4-AP= 4-aminopyridine, Ach = acetylcholine, PE = phenylephrine, X = xanthine, XO = xanthine oxidase.

We next determined whether ACh-induced vasodilatation in the carotid artery was mediated by NOS-generated ROS, focusing on superoxide anion (O_2_^−^), hydrogen peroxide (H_2_O_2_, the more stable product of O_2_^−^), or peroxynitrite (ONOO^−^, the product of the reaction of NO with O_2_^−^). Carotid arteries were incubated with tempol (an O_2_^−^ scavenger), catalase (an enzyme responsible for catalyzing H_2_O_2_ decomposition), or ebselen (an ONOO^−^ scavenger) before examining ACh vasodilatory responses. Ebselen and, to a lesser extent, tempol impaired ACh-induced vasodilatation in the carotid arteries of control and *db/db* mice (Figure 3a), suggesting a role for ONOO^−^ in mediating ACh-induced vasodilatation in carotid arteries. 

#### 2.4.2. Effect of Exogenous O_2_^−^ or ONOO^−^ on Pre-Constricted Aortae and Carotid Arteries

To confirm the vasorelaxant effect of ONOO^−^ in the carotid artery, we exposed pre-constricted carotid arteries to an exogenous ONOO^−^-generating system (xanthine, xanthine oxidase, and a low concentration of SNP). Adding xanthine and xanthine oxidase, with or without SNP, relaxed the carotid artery (51.78 ± 6.72%) (Figure 3b). Adding either xanthine or xanthine oxidase alone did not cause vasodilatation, ruling out nonspecific effects and suggesting that vasodilatation observed in carotid arteries was mediated by O_2_^−^ or O_2_^−^ metabolite(s); O_2_^−^ reacts with basal nitric oxide (NO). This vasorelaxant effect of xanthine and xanthine oxidase in the carotid artery was inhibited by 4-aminopyridine (4-AP) (Figure 3c), suggesting a role for K_v_ in mediating the vasodilatation. This treatment (xanthine + xanthine oxidase) did not relax the aorta, but adding ACh later caused vasodilation in the aorta (Figure 3d).

#### 2.4.3. Effects of Potassium Channel Inhibitors on Endothelium-Dependent Vasodilatation in the Carotid Artery

**Figure 4:**
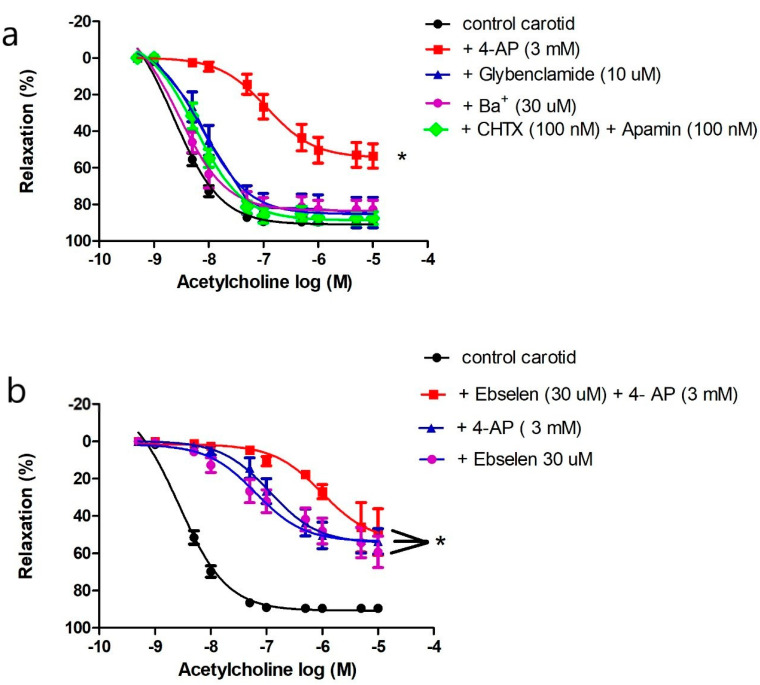
Role of potassium channels in mediating acetylcholine-induced vasodilatation in the carotid artery. (**a**) Cumulative concentration–response curves of acetylcholine in the carotid arteries of control mice (14–20 weeks old) in the presence or absence of 4-aminopyridine, glybenclamide, barium, or the combination of charybdotoxin and apamin (*n* = 5–10 mice). (**b**) Cumulative concentration–response curves of acetylcholine in the carotid arteries of control mice (14–20 weeks old) in the presence or absence of 4-aminopyridine, ebselen, or their combination (*n*= 5–10 mice). Results are displayed as mean ± SE. Dose–response curves were compared by two-way repeated-measures ANOVA followed by Bonferroni post-test. ACh Emax was compared by one-way ANOVA. * denotes *p* < 0.05 vs. control carotid. 4-AP = 4-aminpyridine, CHTX = charybdotoxin.

ROS regulates vascular tone by interacting with potassium channels [32,34,35]. Four types of potassium channels are expressed in the vasculature. We preincubated carotid arteries with 4-AP to inhibit K_V_, glybenclamide to inhibit K_ATP_, barium chloride to inhibit K_ir_, or a combination of charybdotoxin and apamin to inhibit K_Ca_. Only 4-AP impaired ACh-induced vasorelaxation in carotid arteries (Figure 4a). 

We compared the effects of 4-AP, ebselen, or their combination on ACh-induced vasorelaxation to examine if K_V_ is the target of ONOO^−^ in carotid arteries. Addition of 4-AP, ebselen, or their combination reduced ACh maximum response to the same magnitude (Emax: 53.59 ± 2.67%, 47.09 ± 3.57%, 48.34 ± 5.06% respectively, *p* > 0.05) (Figure 4b); there was no additive effect suggesting that ONOO^−^ and K_v_ are components of the same pathway. Additionally, the vasorelaxant effect of xanthine and xanthine oxidase in the carotid artery was inhibited by 4-AP as described above (Figure 3c), confirming a role for K_v_ in mediating carotid artery vasodilatation under physiological and pathological conditions of oxidative stress.

### 2.5. Generalized Vascular Dysfunction in the Aortae of db/db Mice

#### 2.5.1. Effects of Indomethacin, Apocynin, L-NAME, and Tempol on Impaired ACh-Induced Vasodilatation in the Aortae of *db/db* Mice

**Figure 5:**
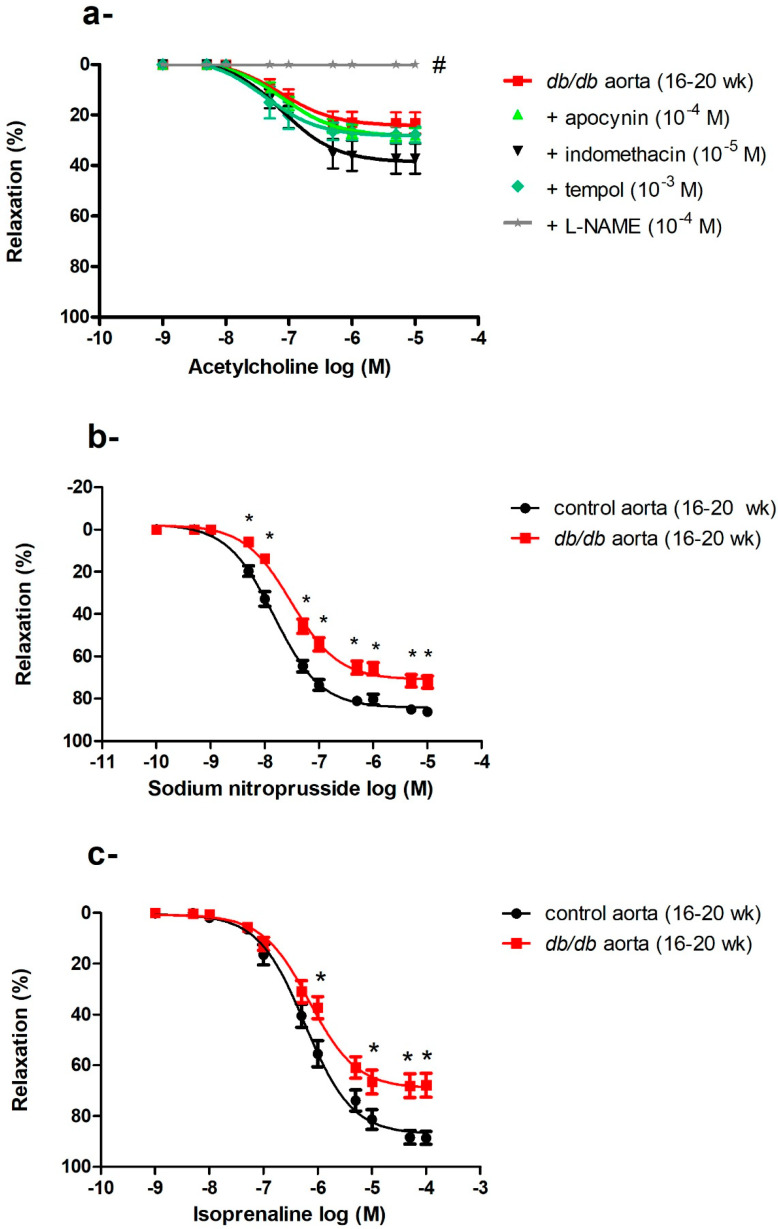
Generalized vascular dysfunction in in the aortae of *db/db* mice. (**a**) Effects of apocynin, indomethacin, L-NAME, and tempol on ACh-induced vasodilatation in the aortae of *db/db* mice at 16–20 weeks of age (*n* = 5–10 mice). (**b**) Cumulative concentration–response curves of sodium nitroprusside in the aortae of *db/db* mice at 16–20 weeks of age (*n* = 6–10 mice). (**c**) Cumulative concentration–response curves of isoprenaline in the aortae of *db/db* mice at 16–20 weeks of age (*n* = 5 mice). Values are displayed as mean ± SE. Dose–response curves were compared by two-way repeated-measures ANOVA followed by Bonferroni post-test. Emax was compared by one-way ANOVA. * denotes *p* < 0.05 vs. control aorta (16–20 weeks), # denotes *p* < 0.05 vs. *db/db* aorta (16–20 weeks).

ROS can be generated from various sources in the vasculature, including NADPH oxidase, uncoupled eNOS, COX, and respiratory chain enzymes [27]. We reasoned that inhibiting the enzyme producing the greatest output of ROS in the aorta would result in the largest improvement in ACh-induced vasodilatation. The aortae of *db/db* mice (16–20 weeks old) were preincubated with an NOS inhibitor (L-NAME), a COX inhibitor (indomethacin), an NADPH oxidase inhibitor (apocynin), or a cell-permeable SOD mimetic (tempol) before examining ACh-induced vasodilatation. None of these treatments, including tempol (at a dose that caused maximum suppression of the O_2_^−^ level in cultured VSMCs [36]), improved ACh-induced vasodilatation. L-NAME abolished the ACh-induced vasodilatation (Figure 5a).

#### 2.5.2. Impaired Endothelium-Independent Vasodilatation in the Aortae of *db/db* Mice

The aortae of *db/db* mice showed an age-dependent progressive deterioration in endothelium-dependent, ACh-induced vasodilatation as described above. We then examined endothelium-independent vasodilatation by using SNP, a direct NO donor that bypasses the endothelium and acts directly on VSMCs. There was no difference in SNP-induced vasodilatation between control and *db/db* mice aged 6–8 weeks old, but the responses to SNP were blunted in 16–20-week-old *db/db* mice (Emax: control _(16–20 weeks)_ = 84.38 ± 1.04% vs. *db/db*_(16–20 weeks)_ = 71.0 ± 1.25%, *p* < 0.05) as shown in Figure 5b. To investigate if vascular dysfunction was confined to the NO/cyclic guanine monophosphate (cGMP) mediated pathway, we examined the response to isoprenaline, a β-adrenoceptor agonist that acts via the cyclic adenosine monophosphate (cAMP) pathway [37]. A reduced response to isoprenaline was also observed in the aortae of *db/db* mice (Emax: control _(16–20 weeks)_ = 87.21 ± 1.83% vs. *db/db*_(16–20 weeks)_ = 69.19 ± 2.02%, *p* < 0.05) as shown in Figure 5c.

### 2.6. Impaired Vasodilatation in Femoral Arteries of db/db Mice 

**Figure 6:**
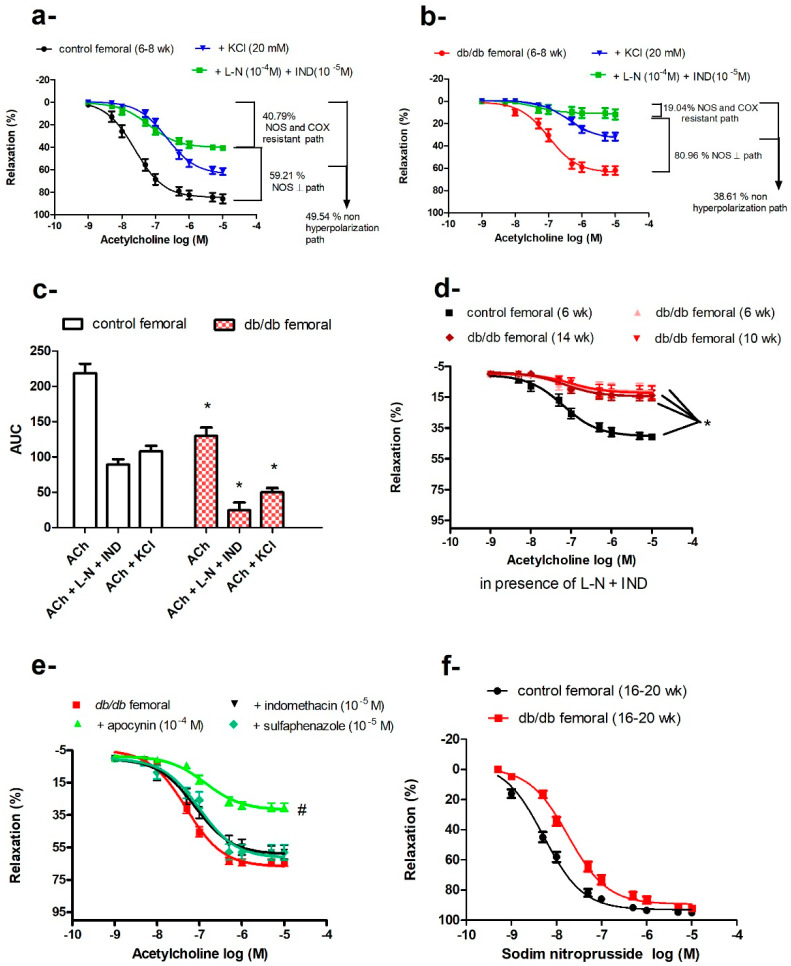
Impaired vasodilatation in the femoral arteries of *db/db* mice. Cumulative concentration–response curves of acetylcholine in the femoral arteries of control mice (**a**) and *db/db* mice (**b**) at 6–8 weeks old in the presence or absence of L-NAME and indomethacin or KCl (*n* = 7–10). (**c**) Relative contribution of nitric oxide synthase (NOS)- and cyclooxygenase (COX)-resistant component and KCl-resistant component to acetylcholine-induced vasodilatation in control and *db/db* mice, as reflected by their respective area under the curve (AUC). (**d**) Cumulative concentration–response curves of acetylcholine in the presence of L-NAME and indomethacin in the femoral arteries of control and *db/db* mice at 6–8 weeks, 10–14 weeks, and 16–20 weeks of age (*n* = 7–10 mice). (**e**) Effects of apocynin, indomethacin, and sulfaphenazole on acetylcholine-induced vasodilatation in the femoral arteries of *db/db* mice at 16–20 weeks of age (*n* = 5–10 mice). (**f**) Cumulative concentration–response curves of sodium nitroprusside (SNP) in the femoral arteries of *db/db* at 16–20 weeks old (*n* = 6–10 mice). Values are displayed as mean ± SE. Dose–response curves were compared by two-way repeated-measures ANOVA followed by Bonferroni post-test. Areas under the curve were compared by two-way ANOVA followed by Bonferroni post-test. * denotes *p* < 0.05 vs. control femoral, # denotes *p* < 0.05 vs. *db/db* femoral. AUC = area under the curve, COX = cyclo-oxygenase, IND = indomethacin, L-N = L-NAME, NOS = nitric oxide synthase.

Vasodilatory responses to ACh were impaired in the femoral arteries of *db/db* mice; this reduced response started at 6–8 weeks of age and did not further deteriorate with age in contrast to age-dependent losses in the aortae (Figure 1d,f). To elucidate the ACh signaling pathways in the different arteries, we preincubated arteries with indomethacin, a combination of L-NAME and indomethacin, a depolarizing concentration of KCl, or a combination of L-NAME, indomethacin, and KCl. Indomethacin did not affect ACh-induced vasodilatation in *db/db* or control mice. Preincubation with L-NAME plus indomethacin reduced ACh-induced vasodilatation in the femoral arteries but did not abolish it, in contrast to the aortae and carotid arteries, where L-NAME abolished the vasodilatation. This residual vasodilatation in the femoral arteries was abolished by KCl or the combination of charybdotoxin and apamin, suggesting that this component of ACh-induced vasodilatation was mediated by an endothelium-dependent hyperpolarizing factor (EDHF). Both NOS- and EDHF-mediated pathways were impaired in *db/db* mice (Figure 6a,b).

The contribution of the EDHF-mediated component, compared with the NOS-mediated component, was more markedly reduced in *db/db* mice, as reflected by reduced AUC_EDHF_ (*db/db* = 24.72 ± 10.89 area units, 19.04% vs. control = 89.22 ± 7.65 area units, 40.79%, *p* < 0.05). The residual AUC remaining after subtracting the component attributed to EDHF should reflect the NOS-dependent component (AUC_NOS_), which was also reduced in *db/db* mice (105.08 area units) vs. control (129.48 area units). However, the relative contribution of the NOS-mediated component to total ACh-induced vasodilatation was higher in *db/db* (80.96%) compared with control mice (59.21%), as shown in Figure 6c. Isolating the NOS-dependent component by using KCl to eliminate the EDHF-dependent pathway showed that this depolarization-resistant pathway was also reduced in *db/db* mice (AUC_KCl resistant_: *db/db* = 50.12 ± 5.84 area units vs. control = 108.21 ± 7.73 area units, *p* < 0.05). 

The sum of AUCs yielded by the KCl-resistant component plus NOS- and COX-resistant component was smaller than the total AUC of ACh-induced vasodilatation. This difference was even more pronounced in *db/db* mice; AUC_KCl resistant_ (50.12 ± 5.84) + AUC_L−NAME and indomethacin resistant_ (24.72 ± 10.89) < AUC_ACh_ (129.8 ± 11.84), suggesting that NO and EDHF have synergistic actions. As mentioned previously, ACh-induced vasodilatation in femoral arteries of *db/db* mice did not deteriorate with age, as was the case of EDHF-mediated vasodilatation (Figure 6d). 

To identify the major enzymatic system(s) contributing to impaired ACh-induced vasorelaxation in *db/db* femoral arteries (16–20 weeks old), we pre-incubated the femoral arteries with indomethacin, apocynin, or a cytochrome P450 inhibitor (sulfaphenazole) before examining the responses to ACh. None of these treatments improved the vasodilatation in *db/db* femoral arteries and, in fact, apocynin further reduced ACh-induced vasodilatation (Figure 6e). From 16–20 weeks of age, femoral arteries of *db/db* mice exhibited reduced sensitivity to SNP (−log EC_50_: *db/db*_(16–20 weeks)_ = 7.7 ± 0.05 M vs. control _(16−20 weeks)_ = 8.28 ± 0.04 M, *p* < 0.05) suggesting a dysfunction at the level of VSMCs as well (Figure 6f).

### 2.7. Protein Expression of SOD-1, SOD-2, and SOD-3 in the Aortae, as well as Carotid and Femoral Arteries, of db/db and Control Mice

**Figure 7:**
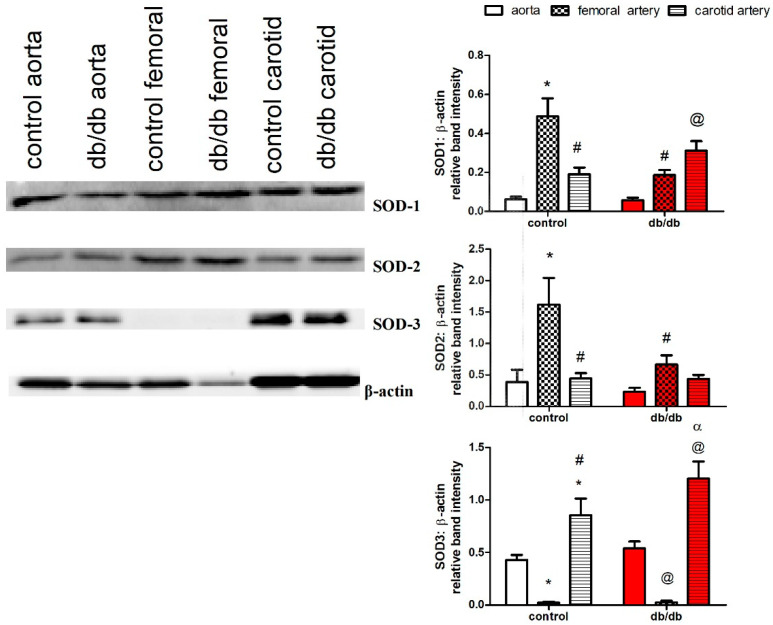
Protein expression of superoxide dismutase (SOD) isoforms in the aortae, as well as carotid and femoral arteries, of *db/db* (red filling) and control mice (white filling) (16–20 weeks old) (*n* = 4). Results are displayed as mean ± SE. Relative band intensities were compared by two-way ANOVA followed by Bonferroni post-test. * denotes *p* < 0.05 vs. control aorta, # denotes *p* < 0.05 vs. control femoral, @ denotes *p* < 0.05 vs. *db/db* aorta, α denotes *p* < 0.05 vs. *db/db* femoral artery. SOD = superoxide dismutase.

We examined protein expression of the three isoforms of SOD (cytosolic (SOD-1), mitochondrial (SOD-2), and extracellular (SOD-3)) in the aortae, as well as carotid and femoral arteries. The femoral arteries of control and *db/db* mice lacked expression of SOD-3, while the carotid arteries had a higher expression of SOD-3 compared to the aortae in control and *db/db* mice. In addition, carotid arteries expressed higher levels of SOD-1 than the aortae under diabetic conditions, while the femoral arteries of *db/db* mice had reduced expressions of SOD-1 and SOD-2 compared to control mice (Figure 7). 

## 3. Discussion

Our study examined the heterogeneity in endothelium-dependent vasodilation in the aortae, as well as the carotid and femoral arteries, in *db/db* mice, a commonly used animal model of type 2 diabetes. The aortae of *db/db* mice exhibited progressive impairment in endothelial and VSMCs function, while the carotid arteries maintained unaltered functional responses, and the femoral arteries showed an early reduced EDHF-mediated vasodilatation that did not further deteriorate with age.

When *db/db* mice were 6–8 weeks old, they were glucose-intolerant, hyperinsulinemic, and heavier in weight, but their fasting plasma glucose levels were not higher than their age-matched controls (Figure 1a,b), suggesting that they were prediabetic, as shown in previous studies [38,39]. 

### 3.1. Aortae

The aortae of *db/db* mice (6–8 weeks old) exhibited a subtle but significant impairment in ACh- but not SNP-induced vasodilatation (Figure 1d), indicating early endothelial dysfunction. Endothelial dysfunction precedes the onset of frank hyperglycemia in animal [40,41] and human studies [42,43], and it is associated with obesity-associated inflammation and vascular insulin resistance [44]. 

The aortae of *db/db* mice showed progressive deterioration in ACh-induced vasodilatation with age (Figure 1d). Obesity, hyperglycemia, and insulin resistance in *db/db* mice could all contribute to endothelial dysfunction in the aortae via an array of mechanisms, with a key role for oxidative stress [27,45,46,47]. ROS can modulate ACh-induced vasodilatation via multiple mechanisms, including receptor binding, eNOS activation, NO bioavailability, activation of soluble guanyl cyclase, and/or interactions with contractile proteins [9,27,48]. There are several enzymatic sources of ROS in the vasculature [49]. Pre-incubation of the aortae of *db/db* mice (16–20 weeks old) with L-NAME, indomethacin, apocynin, or tempol did not improve ACh-induced vasodilatation (Figure 5a), suggesting that either endothelial dysfunction was irreversible in older *db/db* mice (16–20 weeks) or metabolic abnormalities other than oxidative stress contributed to endothelium dysfunction in the aortae of *db/db* mice [25,50,51], a claim that is further supported by our finding that vasodilatory responses to SNP and isoprenaline were also suppressed (Figure 5b,c), suggesting that the dysfunction involved mechanisms beyond scavenging of NO by ROS. This suggestion is also reinforced by findings that incubation with SOD was unable to improve endothelial function in old rats [52], and that catechin (an antioxidant polyphenol) could not improve endothelial function in aged atherosclerotic mice [53]. In addition, treatment with curcumin ameliorated vascular dysfunction in diabetic rats at early but not later stages of diabetes [54]. Importantly, the long-term clinical benefits of traditional antioxidants, e.g., vitamin E, C, or A, in reducing cardiovascular complications were not demonstrated [55,56,57].

To determine if the vascular dysfunction in the aortae of *db/db* mice was limited to the endothelium, we examined the responses to SNP, which releases a NO moiety that does not react with O_2_^−^ [58]. The vasodilatory response to SNP was also impaired in the aortae of *db/db* mice (16–20 weeks old) (Figure 5b). Additionally, the vasodilatory responses to isoprenaline, which are cAMP-mediated [59], were reduced (Figure 5c) consistent with previous studies reporting impaired isoprenaline-induced vasodilatation in diabetic subjects [60,61,62], which was attributed to reduced K_v_ channel activity [62,63]. The ability of ROS to alter vascular Ca^2+^ regulation [64] and potassium channel activity [32], the two major regulators of vascular tone [35], may explain, in part, the progressive deterioration in endothelium-independent vasodilatation in *db/db* mice aortae. Our results suggest that endothelial dysfunction occurs early in diabetes and progresses to VSMC dysfunction at advanced stages. 

### 3.2. Carotid Artery

The responses to ACh were preserved in the carotid arteries of *db/db* mice at the three ages examined (Figure 1e). Although there is evidence of differential adaptation of arteries in aging [52], hypoxia [65], high-fat diet-induced obesity [66], and streptozotocin-induced diabetes [67], few studies explored the mechanisms underlying vascular heterogeneity. We explored the mechanisms underlying the differential adaptation of the aorta and carotid artery in *db/db* mice. The local redox status may be different between the aorta and carotid artery in terms of ROS generation and/or antioxidant defense mechanisms. The production of ROS in the carotid artery might not be higher under diabetic conditions, whereas it is elevated in the aorta. Alternatively, the carotid artery might be able to upregulate its antioxidant defense mechanisms to counterbalance any elevation in local ROS production, thereby maintaining a physiological redox status. A third possibility is that the signaling pathway mediating ACh-induced vasodilatation in the carotid artery is different from that in the aorta and can buffer an increase in ROS levels.

We induced oxidative stress in the carotid arteries and aortae of control mice by using a xanthine/xanthine oxidase O_2_^−^-generating system or by inhibiting SOD, a key antioxidant enzyme, with DCC. The carotid arteries (Figure 2b,d), but not the aortae (Figure 2a,c), relaxed fully to ACh after either intervention, suggesting that carotid arteries are better able to resist oxidative stress. In support of our observation, previous studies reported enhanced O_2_^−^ production in the carotid arteries of *db/db* mice (12 weeks old) [10,68], as well as a greater resistance to oxidative stress in the rabbit carotid artery compared to the aorta [69].

Vasodilatation to ACh was abolished by L-NAME in both aortae and carotid arteries of *db/db* and control mice. However, the maximal response and sensitivity to ACh, but not to SNP, were different in the carotid artery vs. the aorta (Figure 3g,h)*,* suggesting that the ACh signaling pathway in carotid arteries is different from that in the aorta, even though both pathways are NOS-mediated. Since ACh-induced vasodilation in the carotid artery resisted increases in O_2_^−^ production, and since ROS are increasingly recognized as important physiological mediators of vascular tone [70], we examined the roles of O_2_^−^, H_2_O_2_ (a more stable product of O_2_^−^), and ONOO^−^ (the product of the reaction of NO with O_2_^−^, which occurs 3–4 times faster than the dismutation of O_2_^−^ by SOD [71]) in mediating ACh-induced vasodilatation. We incubated carotid arteries with tempol, catalase, or ebselen, and we found that ebselen and, to a lesser extent, tempol impaired ACh-induced vasodilatation in carotid arteries of control and *db/db* mice (Figure 3a), suggesting a key role for ONOO^−^/O_2_^−^ in mediating ACh-induced vasodilatation in carotid arteries. Adding xanthine and xanthine oxidase, with or without SNP, generating O_2_^−^ or ONOO^−^, respectively, relaxed pre-constricted carotid arteries but not aortae (Figure 3b,d), confirming that ONOO^−^/O_2_^−^ contributed to vasodilation in carotid arteries. Preincubation with 4-AP inhibited ACh and xanthine/xanthine oxidase-induced vasodilation in carotid arteries (Figure 3c and Figure 4a), indicating a role for K_V_ channels in mediating vasodilatation. Preincubating carotid arteries with 4-AP, ebselen, or their combination reduced ACh maximum response to the same magnitude with no additive effect (Figure 4b) suggesting that ONOO^−^ and K_V_ were part of the same signaling pathway. These findings can explain, at least partially, the ability of carotid arteries of *db/db* mice to maintain maximal relaxation to ACh under diabetic conditions, as the product(s) of NO with O_2_^−^ is/are able to relax carotid arteries. Additionally, carotid arteries had higher expression of SOD-3 compared to the aortae in control and *db/db* mice. Moreover, carotid arteries expressed higher protein levels of SOD-1 than aortae in *db/db* mice (Figure 7).

Our results extend previous findings reporting ROS as mediators of vasodilation in the vasculature [70,72,73,74,75,76,77]. Some antioxidants were shown to reduce vasodilatation; an oral antioxidant cocktail reduced exercise-induced vasodilatation in the brachial arteries of healthy subjects [78], and ebselen attenuated ACh depressor response in rats [79]. Numerous studies reported that the effects of ROS, including ONOO−, are site-dependent. ONOO− induced vasodilatation in the rabbit internal carotid artery, but not the common carotid artery [80], as well as in rat hindquarters but not pulmonary vasculature [75], and it reduced hindquarter and mesenteric but not renal vasculature resistances [74]. Furthermore, ONOO− reduced isoprenaline-induced relaxation in the hindquarters and renal circulation but not in the mesenteric vascular beds [81].

### 3.3. Femoral Artery

The vasodilatory response to ACh in femoral arteries was only partially inhibited by L-NAME, indicating a significant contribution of EDHF to endothelium-dependent vasodilatation (Figure 6a,b), supporting evidence of a more pronounced role for EDHF in smaller vessels [9,67,82]. Similar to the aortae and contrary to the carotid arteries, the femoral arteries of young *db/db* (6–8 weeks old) mice showed an early loss of ACh-induced vasodilatation. EDHF-mediated vasodilatation is impaired in diabetic animals and patients [83]. In *db/db* mice, EDHF was preserved in small mesenteric arteries [84], but attenuated in coronary arterioles [19]. ROS can impair EDHF via numerous mechanisms such as inhibition of intracellular calcium mobilization, inhibition of function and/or expression of the potassium channels, and inhibition of myoendothelial communication via gap junctions [83,85]. However, ACh-induced, EDHF-mediated vasodilatation in the femoral arteries of *db/db* mice did not further deteriorate with age despite concurrent increases in obesity, hyperglycemia, and systemic oxidative stress (Figure 1a,c and Figure 6d). Vasodilatory responses to SNP were also altered in femoral arteries of older *db/db* mice (16–20 weeks old), similar to changes in the aortae (Figure 5b and Figure 6f). Importantly, femoral arteries of control and *db/db* mice lacked expression of extracellular SOD (Figure 7), a finding that needs further study.

Among the three arteries examined, the aorta was the most vulnerable to the diabetic milieu, a finding that may be attributed, at least in part, to the fact that blood flow in the aorta, being the closest to the heart, is highly dependent on cardiac output and, therefore, aortic endothelial cells are subject to high physical stress and pulsatile flow that can increase endothelial cell loss and ignite vascular dysfunction [21]. The carotid artery maintained full relaxation under the diabetic milieu, a finding that may reflect a physiological strategy to maintain blood supply to the brain even under pathological conditions. 

## 4. Materials and Methods

### 4.1. Animals

Male *db/db* mice (BKS.Cg-*m*^+/+^ *Lepr^db^*/J) and their age- and gender-matched normoglycemic heterozygous littermate *db/m* (BKS.Cg-m^+/+^ *Lepr^db^*^/+^/J) controls were purchased from Jackson Laboratory (Bar Harbor, ME, USA). The mice were housed under standard temperature, humidity, and lighting conditions in the animal facility of the University of British Columbia. The mice had free access to standard rodent chow and water. Mice were acclimatized to their housing conditions for one week before the start of experiments when the mice were six weeks old. The mice body weights and fasting blood glucose levels were measured weekly. The protocol for animal care and use was approved by the Animal Care Committee of the University of British Columbia (#A06-0308, approved on 10 November 2006). 

### 4.2. Intraperitoneal Glucose Tolerance Test 

After overnight (9:00 p.m. to 9:00 a.m.) fasting, mice were administered a glucose solution (2.0 g glucose/kg, i.p.). Blood samples were collected from the tail vein at 0, 15, 30, 60, and 120 min after glucose administration. Plasma was separated by centrifugation and stored at −76 °C for later analyses of glucose and insulin by the Glucose Assay kit (Sigma, St Louis, MO, USA) and Mouse Insulin ELISA kit (ALPCO, Salem, NH, USA), following the manufacturers’ instructions.

### 4.3. Plasma and Tissue Sample Collection

Mice (6, 10, or 16 weeks old) were anesthetized with pentobarbital (50 mg/kg, i.p.) combined with heparin (50 U/kg); blood samples were withdrawn from the inferior vena cava and collected into tubes containing 0.005% butylated hydroxy toluene, an antioxidant, to inhibit in vitro formation of ROS. The blood was centrifuged (10 min at 4 °C, 1000× *g*) and the plasma fraction was stored at −76 °C for later measurement of plasma 8-isoprostane levels using an ELISA kit (Cayman Chemical, Ann Arbor, MI, USA). The animals were euthanized by removing the heart after blood collection. The thoracic aortae, as well as the carotid and femoral arteries, were excised and kept in an ice-cold physiologic salt solution (PSS): NaCl (119 mM), KCl (4.7 mM), KH_2_PO_4_ (1.18 mM), MgSO_4_ (1.17 mM), NaHCO_3_ (24.9 mM), EDTA (0.023 mM), CaCl_2_ (1.6 mM), and dextrose (11.1 mM). With the aid of a dissecting microscope and microsurgery instruments, the arteries were rapidly cleared of adherent connective tissues. The dissected arteries were either snap-frozen in liquid nitrogen and stored at −76 °C for Western blot analysis or mounted in wire-myograph chambers for isometric force measurement. 

### 4.4. Assessment of Endothelium-Dependent and-Independent Vasodilatation

Arteries were cut into 2-mm-long rings and mounted in wire-myograph chambers (Danish Myotechnology, Aarhus, Denmark) for isometric force measurement [25,86]. Each vessel chamber was filled with 5 mL PSS that was continually gassed with carbogen (95% O_2_ + 5% CO_2_) and maintained at pH 7.4 and 37 °C. The aortic, carotid, and femoral rings were gradually stretched to their optimal resting tensions (5.5, 4, or 3 mN, respectively) as determined in preliminary experiments. There were no differences in resting tensions of aorta, carotid, and femoral arteries between *db/db* and control mice. The PSS was replaced at 20-min intervals during the adjustment of resting tension; the arterial rings were equilibrated for at least 30 min after reaching their optimum basal tensions; they were then challenged twice with 80 mM KCl before examining the dose–response relationships. 

The aortic or carotid rings were pre-constricted with a submaximal dose (producing 60–80% of the maximum response) of phenylephrine (PE), an α_1_-adrenoceptor agonist. In the femoral arteries, the thromboxane A_2_ analogue U-46619 (10^−8^ to 10^−7^ M) was used as a constrictor because PE did not yield stable contractions. After obtaining a stable contraction, ACh (10^−9^ to 10^−5^ M) was added in half-log increments in a cumulative manner to examine endothelium-dependent vasodilatation. After a 30-min washout period, the arterial rings were re-constricted with PE or U-46619, then SNP (10^−10^ to 10^−5^ M) or isoprenaline (10^−9^ to 10^−5^ M) was added to examine endothelium-independent vasodilatation. In subsets of experiments, arteries were preincubated with different vasoactive agents for 30 min before examining the responses to ACh. The mechanisms and references to the concentration of the vasoactive agents used in the vasoreactivity experiments are listed in Table S1 (Supplementary Materials). Vascular responses were recorded and analyzed by Powerlab 4/25 and Labchart 8 reader (AD Instruments, Sydney, Australia). Percentage relaxation to vasodilators (ACh, SNP, or isoprenaline) was calculated as loss of the initial PE- or U-46619-induced constriction. 

### 4.5. Western Blot

Arteries (aorta, carotid, or femoral) from 3–5 mice (control or *db/db*) were pooled to yield enough protein for Western blot analysis. Frozen arteries were homogenized in ice-cold RIPA buffer (Santa Cruz, Dallas, TX, USA), and the homogenates were centrifuged at 10,000× *g* for 30 min at 4 °C. The protein contents of the supernatants were determined by the Coomassie Plus Protein Assay (Pierce-Thermo Fisher Scientific, Milwaukee, Wisconsin, USA). Protein samples (30–40 μg of total protein) were prepared in Laemmli sample buffer, separated by 8–10% sodium dodecyl sulfate polyacrylamide gel electrophoresis and then transferred overnight (at 4 °C, 40 V) to nitrocellulose membranes. Membranes were then blocked for 1 h in 5% skim milk in Tris-buffered saline containing 0.1% Tween-20, washed (3 × 15 min), and incubated overnight at 4 °C with primary antibodies against SOD-1, SOD-2, SOD-3 (Santa Cruz Biotechnology, Dallas, TX, USA), or β-actin (BD Transduction Labs, Mississauga, ON, Canada) as a housekeeping protein. Membranes were triple-washed and then incubated with their corresponding horseradish peroxidase-conjugated secondary antibodies (Santa Cruz Biotechnology, Dallas, TX, USA) for 2 h. After washing, the blots were visualized using an enhanced chemiluminescent detection kit (Pierce-Thermo Fisher Scientific, Milwaukee, WI, USA) and ChemiDoc XRS (BioRad Laboratories, ON, Canada). Volume analyses of the protein bands were performed by Quantity One software (BioRad Laboratories, Mississauga, ON, Canada).

### 4.6. Statistical Analysis

Results are displayed as means ± standard error (SE). The sample size of each group is specified as (*n*) in the footnote for each figure. Non-linear regression, maximum response (Emax), sensitivity (EC_50_), area under the curve (AUC), and statistical tests were carried out using Prism version 5.0 (GraphPad Software, San Diego, CA, USA). The level of statistically significant difference was set at *p* < 0.05.

## 5. Conclusions

Our study demonstrates substantial heterogeneity and differential adaptation in endothelium- dependent vasodilation in the aortae, as well as the carotid and femoral arteries, from *db/db* mice that could be attributed to differences in signaling pathways and antioxidant enzyme expression. A better understanding of regional vascular heterogeneity will help in the development of targeted vascular treatments, as well as effective strategies for blood vessel replacement to combat severe cardiovascular disease related to diabetes.

## Supplementary Materials

The following are available online at https://www.mdpi.com/1422-0067/21/17/6147/s1, Table S1: Vasoactive agents used in vascular reactivity experiments.
