# Supplementary material for: Redox Signaling and Regional Heterogeneity of Endothelial Dysfunction in db/db Mice
Source: Int J Mol Sci. 2020 Aug 26;21(17):6147. doi: 10.3390/ijms21176147 (PMC7504187; doi:10.3390/ijms21176147)
Supplement: Supplementary file 1 [file ijms-21-06147-s001.pdf]

International Journal of
Molecular Sciences

MDPI

Table 1. Vasoactive agents used in vascular reactivity experiments.

Drug	Mechanism	Concentration	References
4-aminopyridine	Inhibition of K_V	3 x 10^{-3} M	[1,2]
Apocynin	Inhibition of NADPH oxidase	10^{-4} M	[3–5]
Apamin	Blocking of small conductance calcium activated potassium channels	10^{-7} M	[6,7]
Barium chloride	Blocking of inward rectifier potassium channels	3 x 10^{-5} M	[8]
Catalase	Decomposition of hydrogen peroxide	1200 U/mL	[5,8]
Charybdotoxin	Blocking of large and intermediate conductance calcium activated potassium channels	10^{-7} M	[8,9]
Diethyldithiocarbamate	Inhibition of superoxide dismutase	3 x 10^{-3} M	[10,11]
Ebselen	Scavenging of peroxynitrite radical	3 x 10^{-5} M	[12–14]
Glybenclamide	Blocking of K_{ATP}	10^{-5} M	[15,16]
Indomethacin	Inhibition of cyclo-oxygenase	10^{-5} M	[8,17]
L-NAME	Inhibition of nitric oxide synthase	10^{-4} M	[3]
Sulfaphenazole	Inhibition of cytochrome P 450 mono-oxygenase	10^{-5} M	[6,18]
Tempol	Cell permeable superoxide dismutase mimetic	10^{-3} M	[4,5,19]
Xanthine + xanthine oxidase	An exogenous source of superoxide anion	10^{-4} M plus 0.01 U/mL	[9]
Xanthine + xanthine oxidase + a low concentration of SNP	An exogenous source of peroxynitrite radical	10^{-4} M + 0.01 U/mL + 10^{-9} M	[9]

References.

1. Chai, Q.; Lu, T.; Wang, X.L.; Lee, H.C. Hydrogen sulfide impairs shear stress-induced vasodilation in mouse coronary arteries. *Pflugers Arch. Eur. J. Physiol.* **2014**, *467*, 329–340, doi:10.1007/s00424-014-1526-y.
2. Testai, L.; Chericoni, S.; Calderone, V.; Nencioni, G.; Nieri, P.; Morelli, I.; Martinotti, E. Cardiovascular effects of Urtica dioica L. (Urticaceae) roots extracts: In vitro and in vivo pharmacological studies. *J. Ethnopharmacol.* **2002**, *81*, 105–109, doi:10.1016/S0378-8741(02)00055-7.
3. Bagi, Z.; Koller, A.; Kaley, G. PPARgamma activation, by reducing oxidative stress, increases NO bioavailability in coronary arterioles of mice with Type 2 diabetes. *Am. J. Physiol. Heart Circ. Physiol.* **2004**, *286*, H742-8, doi:10.1152/ajpheart.00718.2003.
4. Wong, W.T.; Tian, X.Y.; Xu, A.; Ng, C.F.; Lee, H.K.; Chen, Z.Y.; Au, C.L.; Yao, X.; Huang, Y. Angiotensin II type 1 receptor-dependent oxidative stress mediates endothelial dysfunction in type 2 diabetic mice. *Antioxid. Redox Signal.* **2010**, *13*, 757–768, doi:10.1089/ars.2009.2831.
5. Liang, C.F.; Liu, J.T.; Wang, Y.; Xu, A.; Vanhoutte, P.M. Toll-like receptor 4 mutation protects obese mice against endothelial dysfunction by decreasing NADPH oxidase isoforms 1 and 4. *Arterioscler. Thromb. Vasc. Biol.* **2013**, *33*, 777–784, doi:10.1161/ATVBAHA.112.301087.
6. Otani, S.; Nagaoka, T.; Omae, T.; Tanano, I.; Kamiya, T.; Ono, S.; Hein, T.W.; Kuo, L.; Yoshida, A. Histamine-induced dilation of isolated porcine retinal arterioles: Role of endothelium-derived hyperpolarizing factor. *Investig. Ophthalmol. Vis. Sci.* **2016**, *57*, 4791–4798, doi:10.1167/iovs.15-19038.
7. Randriamboavonjy, V.; Kiss, L.; Falck, J.R.; Busse, R.; Fleming, I. The synthesis of 20-HETE in small porcine coronary arteries antagonizes EDHF-mediated relaxation. *Cardiovasc. Res.* **2005**, *65*, 487–494, doi:10.1016/j.cardiores.2004.10.029.
8. Pannirselvam, M.; Ding, H.; Anderson, T.J.; Triggle, C.R. Pharmacological characteristics of endothelium-derived hyperpolarizing factor-mediated relaxation of small mesenteric arteries from db/db mice. *Eur. J. Pharmacol.* **2006**, *551*, 98–107, doi:10.1016/j.ejphar.2006.08.086.
9. Ohashi, M.; Faraci, F.; Heistad, D. Peroxynitrite hyperpolarizes smooth muscle and relaxes internal carotid artery in rabbit via ATP-sensitive K+ channels. *Am. J. Physiol. Hear. Circ. Physiol.* **2005**, *289*, doi:10.1152/ajpheart.00254.2005.
10. Paravicini, T.M.; Chrissobolis, S.; Drummond, G.R.; Sobey, C.G. Increased NADPH-Oxidase Activity and Nox4 Expression during Chronic Hypertension Is Associated with Enhanced Cerebral Vasodilatation to NADPH In Vivo. *Stroke* **2004**, *35*, 584–589, doi:10.1161/01.STR.0000112974.37028.58.

11. Drouin, A.; Thorin-Trescases, N.; Hamel, E.; Falck, J.R.; Thorin, E. Endothelial nitric oxide synthase activation leads to dilatory H2O2 production in mouse cerebral arteries. *Cardiovasc. Res.* **2007**, *73*, 73–81, doi:10.1016/j.cardiores.2006.10.005.
12. Stinghen, A.E.M.; Chillon, J.-M.; Massy, Z.A.; Boullier, A. Differential effects of indoxyl sulfate and inorganic phosphate in a murine cerebral endothelial cell line (bEnd.3). *Toxins (Basel).* **2014**, *6*, 1742–1760, doi:10.3390/toxins6061742.
13. Müller, A.; Cadenas, E.; Graf, P.; Sies, H. A novel biologically active seleno-organic compound-1. Glutathione peroxidase-like activity in vitro and antioxidant capacity of PZ 51 (Ebselen). *Biochem. Pharmacol.* **1984**, *33*, 3235–3239, doi:10.1016/0006-2952(84)90083-2.
14. Kurozumi, R.; Tokuzumi, S.; Kojima, S. Does peroxynitrite involve in the elevation of cellular glutathione induced by sodium nitroprusside (SNP) in RAW 264.7 cells? *Biol. Pharm. Bull.* **2003**, *26*, 1070–1075, doi:10.1248/bpb.26.1070.
15. Pradhan, I.; Ledent, C.; Mustafa, S.J.; Morisseau, C.; Nayeem, M.A. High salt diet modulates vascular response in A2AAR+/+ and A2AAR−/− mice: Role of sEH, PPARγ, and KATP channels. *Mol. Cell. Biochem.* **2015**, *404*, 87–96, doi:10.1007/s11010-015-2368-4.
16. Tong, X.-K.; Nicolakakis, N.; Fernandes, P.; Ongali, B.; Brouillette, J.; Quirion, R.; Hamel, E. Simvastatin improves cerebrovascular function and counters soluble amyloid-beta, inflammation and oxidative stress in aged APP mice. *Neurobiol. Dis.* **2009**, *35*, 406–414, doi:10.1016/j.nbd.2009.06.003.
17. Leo, C.H.; Joshi, A.; Woodman, O.L. Short-term type 1 diabetes alters the mechanism of endothelium-dependent relaxation in the rat carotid artery. *Am. J. Physiol. Hear. Circ. Physiol.* **2010**, *299*, doi:10.1152/ajpheart.01197.2009.
18. Morio, Y.; Homma, N.; Takahashi, H.; Yamamoto, A.; Nagaoka, T.; Sato, K.; Muramatsu, M.; Fukuchi, Y. Activity of endothelium-derived hyperpolarizing factor is augmented in monocrotaline-induced pulmonary hypertension of rat lungs. *J. Vasc. Res.* **2007**, *44*, 325–335, doi:10.1159/000101778.
19. Luo, Z.; Chen, Y.; Chen, S.; Welch, W.J.; Andresen, B.T.; Jose, P.A.; Wilcox, C.S. Comparison of inhibitors of superoxide generation in vascular smooth muscle cells. *Br. J. Pharmacol.* **2009**, *157*, 935–943, doi:10.1111/j.1476-5381.2009.00259.x.
